# An exploration of New Zealand mental health nurses’ personal physical activities

**DOI:** 10.1111/inm.12981

**Published:** 2022-02-14

**Authors:** Glen Philbrick, Nicolette Fay Sheridan, Kay McCauley

**Affiliations:** ^1^ School of Nursing Massey University Wellington New Zealand

**Keywords:** health promotion, mental health, nurses, physical activity, physical inactivity

## Abstract

This study assessed the physical activities of Mental Health Nurses (MHN) in New Zealand against the 2018 World Health Organization recommended minimum levels of moderate‐to‐vigorous physical activity. The research design was exploratory and descriptive as there were no previous studies about physical activity levels of MHNs in New Zealand. Quantitative and qualitative data were collected using the International Physical Activity Questionnaire (IPAQ, Long Version) which included options for free‐text responses. Data were analysed using descriptive and inferential statistics. A total of 266 participants returned the survey, a response rate of 4%, and a limitation of the study. More than 50% of MHNs reported <150 min of moderate‐to‐vigorous exercise per week for each of the four physical activity domains. When individual physical activity domains were combined, only 10% spent <150 min on moderate‐to‐vigorous physical activity. Work‐related physical activities were higher for those working in the inpatient area than in community settings. Transport‐related physical activities were higher for those working in community settings. Participants registered from 6 to 20 years had more time sitting than other groups. Nurses aged 55 years and above showed the highest total physical activity levels. Moreover, healthcare organizations and nurse leaders need to promote physical activity and provide wellness intervention for their staff. Nurses who are physically active may be more effective in supporting their patients to increase their physical activity.

## Introduction

World Health Organization guidelines recommend that every individual should undertake at least 150 min of moderate or vigorous physical activity per week (World Health Organization 2018). Physical inactivity is a major global health pandemic and one of the leading causes of morbidity and premature mortality worldwide (Blake *et al*. 2017; Hafner *et al*. 2020; Lee *et al*. 2012; Lobelo *et al*. 2014). Global physical inactivity levels in some countries are up to 70%, due to changing transport patterns, increased digital and technological communication, and urbanization (World Health Organization 2018). In 2016, the World Health Organization estimated that the global prevalence of physical inactivity was 27.5%; however, over the last two decades, this prevalence has ranged between 23% and 32% due to variations in measurement methods (Guthold *et al*. 2018; WHO 2018).

There is overwhelming evidence that physical inactivity is linked to an increased risk of non‐communicable diseases including, coronary heart disease, type 2 diabetes, stroke, hypertension, and several cancers (Beighton *et al*. 2015; Friedenreich *et al*. 2021; Hallal *et al*. 2012; Laeremans *et al*. 2017; Lobelo & de Quevedo 2016; Silva *et al*. 2018). Physical inactivity is also associated with non‐communicable disease risk factors such as hypertension, overweight, and obesity (Faruque *et al*. 2021) and a higher risk for severe COVID‐19 outcomes (Sallis *et al*. 2021). Of particular concern is 7.2% and 7.6% of non‐communicable diseases and cardiovascular deaths globally are attributed to physical inactivity (Katzmarzyk *et al*. 2021). Globally, 7.2% and 7.6% of all‐cause and cardiovascular disease deaths, respectively, are attributable to physical inactivity.

The financial cost of global physical inactivity to the world healthcare system in 2013 was estimated at US$53.8 billion dollars (Ding *et al*. 2016). This estimate was retrieved from direct healthcare costs, productivity costs, and disability‐adjusted‐life costs associated with the global physical inactivity levels from 142 countries, comprising 92.1% of the world’s total population (Ding *et al*. 2016).

## Background

### Physical inactivity in registered nurses

There is a paucity of research on registered mental health nurses’ (MHNs) physical activity levels in New Zealand. However, there have been numerous studies investigating the physical activity levels of registered nurses globally. Previous research has revealed that nurses’ participation rate in physical activity is among the lowest compared with other professions (Chan & Perry 2012). Jirathananuwat and Pongpirul (2017) conducted a cross‐sectional survey and compared the physical activity levels of 142 nurse clinical practitioners with 147 nurse managers during working hours. The average moderate‐to‐vigorous physical activity levels for all nurse clinical practitioners and nurse managers were 113.12 min per week, which did not meet the World Health Organization (2018) mandated physical activity‐level guidelines, which were current at the time of the study. Reed *et al*. (2018) undertook a multi‐centre cross‐sectional study across 14 hospitals to assess the physical activity levels of 410 nurses. Only 23% of nurses met the World Health Organization (2018) minimum recommended daily levels of physical activities of ‘at least 150 min per week of moderate‐to‐vigorous intensity physical activity’. The collective evidence from the literature suggests that nurses are not achieving the World Health Organization (2018) minimum recommended daily levels of physical activities (Bakhshi *et al*. 2015; Chappel *et al*. 2017; Flannery *et al*. 2014; Jirathananuwat & Pongpirul 2017; McCarthy *et al*. 2018; Reed *et al*. 2018; Ross *et al*. 2017; Torquati *et al*. 2017).

### Occupational physical activity levels

Different types of physical activity are defined as any bodily movement produced by skeletal muscles that requires energy expenditure. Physical activity refers to all movement including during leisure time, for transport to get to and from places, or as part of a person’s work. Both moderate‐intensity physical activity and vigorous‐intensity physical activity improve health (World Health Organization 2020).

Several studies have reported that nurses’ occupational physical activity levels were found to be of light to moderate intensity and not meeting the moderate‐to‐vigorous physical activity‐level recommendations (Chappel *et al*. 2017; Lee *et al*. 2005). These findings have been confirmed by several studies, including an Australian study by Zhao *et al*. (2012), which revealed that 50% of nurses reported low occupational physical activity levels, which did not meet the minimum levels of physical activity recommendations.

Moreover, despite studies showing nurses walking up to five miles during a 10‐h shift, most nurses do not meet the minimum global moderate‐to‐vigorous physical activity levels (McCarthy *et al*. 2018).

Conversely, a study from the United States of America of exercise habits of nurses conducted by Flannery *et al*. (2014) found that 76.9% of 71 nurses achieved the national minimum guidelines for exercise (300 min of exercise per week). However, the authors suggested that the participants over‐reported their physical activity levels.

### Sedentary behaviour

Over the last decade, there has been an exponential growth in the research of ‘sedentary behaviour’ (and the potentially detrimental effects on health). Sedentary behaviour ‘comprises a set of waking time activities characterized by an energy expenditure of ≤1.5 metabolic equivalents in a sitting or reclining posture’ (Sedentary Behaviour Research Network 2012; Tremblay *et al*. 2017). Numerous studies have reported that on workdays, nurses spend more than fifty per cent of their time being sedentary and very few attain current physical activity recommendations (Prince *et al*. 2019; Ratner & Sawatzky 2009; Reed *et al*. 2018). Prince *et al*. (2019) revealed that nurses spend 7.5 waking hours/day being sedentary. Similar results demonstrated that public health nurses remained seated without movement for over 8 h per day (Lin *et al*. 2018).

These findings are concerning as there is mounting evidence of a greater risk of cardiometabolic disease and mortality when sedentary time exceeds 8 h daily (Ekelund *et al*. 2016; Prince *et al*. 2019).

### Contributing factors to low physical activity levels

Contributing factors to low physical activity levels include overwork, irregular shifts, and stress (Torquati *et al*. 2017). Other barriers include the nurse’s age, poor self‐care, beliefs about exercise, lack of time, inadequate support, limited access to exercise facilities, and decentralized nurses’ stations, resulting in nurses walking shorter distances (Al‐Kandari *et al*. 2008; Reed *et al*. 2018; Ross *et al*. 2017).

Consequently, there is an urgent need for strategies to increase nurses’ physical activity levels and mitigate the risk of nurses developing NCDs (Torquati *et al*. 2017).

### Nurse’s role in health promotion

Health professionals can play a key role in overcoming the current global pandemic of physical inactivity (Sundberg 2016). Several studies confirmed that nurses who engage in health‐promoting physical activity are better role models and advocates for promoting health in their consumers (Bakhshi *et al*. 2015; Esposito & Fitzpatrick 2011). Stanton *et al*. (2015) argued barriers for nurses to provide exercise counselling included their low confidence to prescribe exercise, lack of time, and lack of training. Duignan and Duignan (2017) recommended all emergency nurses are trained in motivational interviewing for the purpose of promoting physical activity and exercise with their consumers.

It is concerning that the rate of physical inactivity in individuals diagnosed with serious mental illness was greater than the general population (Happell *et al*. 2012; Rosenbaum *et al*. 2016). The mental health nursing workforce is strategically placed to educate, empower, and implement physical activity counselling and interventions for mental health consumers (Happell *et al*. 2012). However, to the best of our knowledge, no study has investigated the physical activity levels of mental health nurses (MHNs).

This study aimed to examine the physical activities of MHNs in New Zealand and whether they achieved WHO recommended minimum levels of moderate‐to‐vigorous physical activity (World Health Organization 2018).

## Method

### Design

The research design was exploratory and descriptive as there were no previous studies about physical activity levels of MHNs in New Zealand. The International Physical Activity Questionnaire (IPAQ), questions about demographics (gender, age, employment setting, years of nursing practice experience, ethnicity), and an open text response question, were sent via an email link to all known MHNs in NZ in 2018.

### Participants

Potential participants were identified from three membership bodies: New Zealand College of Mental Health Nurses (NZCMHN, *n = *280), New Zealand Nurses Organisation (NZNO, *n = *752), and Public Service Association (PSA, *n = *5630).

In 2018, the researcher sent a letter to the president of each of the membership bodies requesting assistance with recruitment. Each president agreed to email all members a cover letter, Participant Information Sheet, and flyer with an anonymous SurveyMonkey link to the IPAQ. The Participant Information Sheet included an overview and guide to the IPAQ.

### Data collection

The IPAQ (Long Version) instrument (Craig *et al*. 2003) **is** open access. The instrument was approved as culturally safe, without changes, by the Māori Health Development Unit in an urban city hospital. The key feature of the IPAQ is ‘its ability to provide, in detail, participation estimates for multiple domains of physical activity, including leisure‐time physical activity, physical activity for transportation, physical activity in the home and physical activity at work’ (Sebastiao *et al*. 2012 p. 968).

Each domain includes three categories: ‘walking (W)’, ‘moderate‐intensity (M)’, and ‘vigorous‐intensity activity (V)’. These categories are scored from duration (minutes) and frequency (days) when reporting physical activity levels data outcomes (IPAQ Group 2015).

The category scores were defined as high, medium, and low physical activity for each of the above four activity domains based on guidelines for ≥150 and <150 min per week for all activities lasting longer than 10 min each time (Lear *et al*. 2017). Total weekly levels of moderate‐intensity physical activity were measured.

The IPAQ is the most widely used instrument to collect physical activity level information from a population (Silva *et al*. 2017). Because the IPAQ is a self‐report instrument, it may be subject to bias and inaccuracies, and previous studies have shown conflicting results between the IPAQ and accelerometers (Silva *et al*. 2017).

Some researchers advise combining the IPAQ with an accelerometer to avoid self‐report bias (Silva *et al*. 2017). However, the use of accelerometers was beyond the scope of this study. Other researchers have found the IPAQ to have sufficient test–retest reliability (Blake *et al*. 2017).

The IPAQ produced repeatable data as evidenced by the correlation coefficient score of 0.8 across 12 countries (Craig *et al*. 2003). The criterion validity had a median *ρ* of 0.30 and was comparable to other self‐report validation studies.

### Ethics approval

This study obtained approval from the Massey University Ethics Committee and complied with the Massey University Code of Ethical Conduct (Massey University, 2017). This study was identified as ‘low risk’. Approval ID Number 4000017802.

### Informed consent

This research project ensured all participants had sufficient information before the study to provide informed consent without feeling coerced or made to feel obliged to participate.

The participant information sheet informed potential participants that they could withdraw from the online survey before submitting their questionnaire responses. Once submitted, participants could not withdraw because all responses were anonymous.

### Statistical analysis

All online survey questionnaire responses were analysed using descriptive and inferential statistics.

The data were analysed using the IBM SPSS Statistics version 24.0 (IBM Corp 2016) and R software (ver. 3.5.1) (R Core Team 2013). The descriptive statistics were used to summarize demographic data. Due to the small counts in some demographic data groups, data were merged. The nonparametric alternative methods used include the Wilcoxon rank‐sum test and the Kruskal–Wallis rank‐sum test. The Wilcoxon rank‐sum test is used when the data are not normally distributed (DePoy & Gitlin 2015). The Kruskal–Wallis test evaluated the differences among groups by estimating rank differences among them. If the Kruskal–Wallis test is significant, then a post hoc test is conducted using the Wilcoxon rank‐sum test, which then investigates which groups differ significantly, using pairwise comparisons.

## Results

### Demographic data of survey participants

Responses were received from 266 MHNs. Of the 266 participants, 200 (75%) were female (Table 1). Forty‐five per cent of participants were 35–54 years of age, and 38% were 55 years and above. Most were NZ European/Pakeha (74%) with 15% Other Europeans. New Zealand Maori were 3.1% and Pacific peoples 12.6%. All other ethnic groups comprised 5.7% of the study population. The mental health employment settings primarily included community comprised of 60% male and female MHN’s. Inpatient employment settings included 34.8% male and 33% female MHN’s. The number of years participants were registered as a MHN included <12 months (5%), 1–5 years (17%), 6–11 years (13%), 12–20 years (21%), 21–30 years (21%), and 31 years or greater (23%).

**Table 1:** Demographic data of survey participants

Demographic variable	Groups Gender	Male	Female
Age	18‐24 years	0.0%	3.0%
	25‐34 years	9.0	16.5
	35‐54 years	43.9	46.5
	>55 years	47	34.0
Ethnicity	NZ European/Pakeha	68.3	75.3
	Other European	20.0	13.6
	NZ Maori	0.0	3.1
	Cook Island Maori	1.7	0.6
	Tongan	1.7	0.6
	Niuean	1.7	0.0
	Tokelauan	3.3	1.8
	Fijian	0.0	0.6
	Other Pacific Peoples	0.0	0.6
	Indian	3.3	1.2
	Other Asian	0.0	0.6
	African	0.0	0.6
Mental health clinic setting	Inpatient	34.8	33
	Community	60.0	60
	Nursing Education	1.5	2.0
	Nursing Management	4.5	3.0
	Nursing Policy & Research	0.0	1.5
Years of registration	< 12 months	1.6	5.6
	1–5 years	9.5	19.8
	6–11 years	11.1	13.2
	12–20 years	19.0	22.3
	21–30 years	28.57	19.29
	31 years +	30.16	19.80

### Total weekly mean minutes of work‐related physical activity

**Fig. 1:**
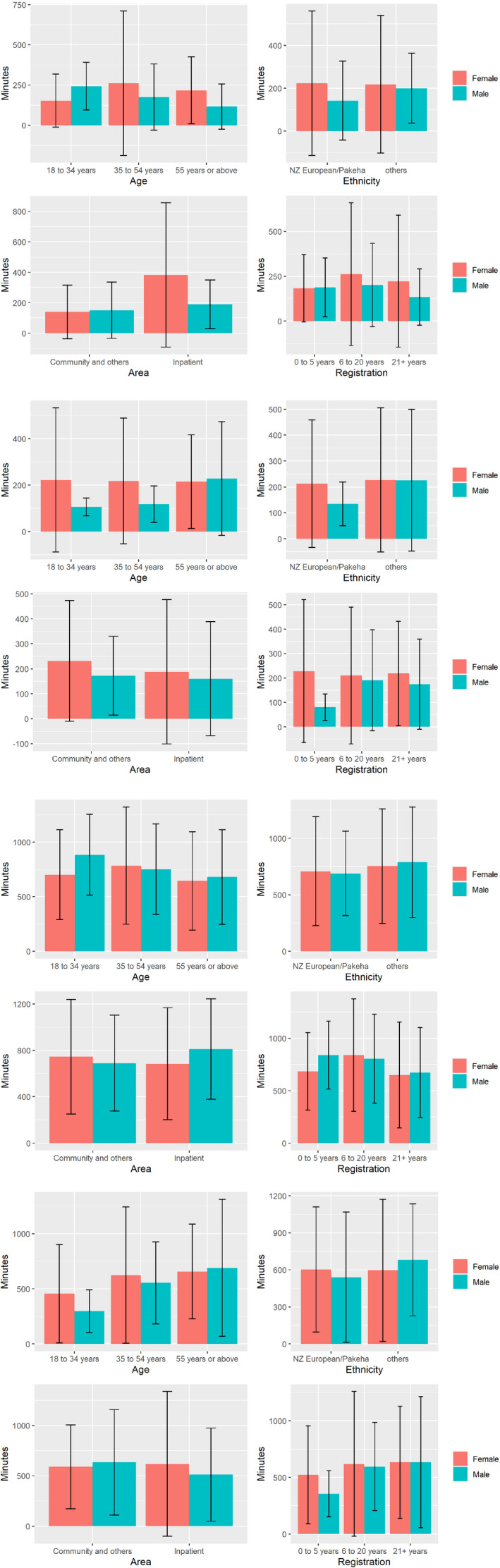
Graphical display of mean total minutes of physical activities weekly using bar plots shows the distributions of the total weekly minutes of work, transport, sitting behaviours, and total physical activities (leisure and household physical activity levels were not specifically reported in this study, but were included in the total physical activity calculation). Vertical lines in the centre of each blue bar are error bars. Error bars shows how precise a measurement is. The lower limit of the error bar is equal mean value (top of the bar) minus standard deviation (one or two). The upper limit of the error bar is equal mean value plus standard deviation (one or two). Bar plots show mean +/‐ 2 standard deviations. (1.1) Total weekly mean minutes of work‐related physical activity by gender, age ethnicity, area of work, and years of registration. (1.2) Barplots of Total Weekly Mean Minutes of Transport‐related Physical Activity by gender, age ethnicity, area of work, and years of registration. (1.3) Barplots of Total Mean Minutes Spent Sitting by gender, age ethnicity, area of work, and years of registration. (1.4) Barplots of Mean total weekly minutes of all physical activities by gender, age ethnicity, area of work, and years of registration.

Figure 1 shows no statistically significant associations between total time for work‐related physical activity and sociodemographic variables.

### Nonparametric results for total weekly minutes of work‐related physical activity

The Wilcoxon rank‐sum test results for total weekly minutes of work‐related physical activity (Table 2) demonstrated that participants working in inpatient areas of mental health (median = 240) scored higher on work‐related physical activity than participants in the community or other areas of mental health (median = 70).

**Table 2:** Wilcoxon rank‐sum test results for total weekly minutes of job‐related physical activity

Variable/Group	*n*	Median	IQR	Mean	Mean rank	*W* statistic	df	*P*‐value
Gender						1700	1	0.759
Female	199	90	230	221	130			
Male	66	90	150	160	147			
Ethnicity						2249	1	0.477
NZ European	164	80	200	203	133			
Others	103	100	235	215	135			
Area						1347	1	0.0002
Community & others	178	70	148	141	128			
Inpatient	89	240	390	352	146			

The Wilcoxon rank‐sum test *W*‐value was statistically significant (*W* = 1347, *P* = 0.0002). The Kruskal–Wallis test results in Table 3 showed no significant difference in the mean ranks between the three age groups (*H* (2) = 0.747, *P* = 0.688).

**Table 3:** Kruskal–Wallis test results for total weekly minutes of job‐related physical activity

Variable/Group	*n*	Median	IQR	Mean	Mean rank	H statistic	df	*P*‐value
Age						0.747	2	0.688
18–34 years	45	100	165	163	119			
35–54 years	122	75	218	241	135			
55 years or above	100	90	240	191	140			
Registration						0.486	2	0.784
0–5 years	57	100	270	183	112			
6–20 years	121	75	260	250	145			
21+ years	89	90	152	195	136			

### Total weekly mean minutes of transport‐related physical activity

The mean total weekly minutes of transport‐related physical activity revealed no associations between sociodemographic variables on the mean total times for transport‐related physical activity levels.

The error bars for all categories were sizeable and overlapping. Therefore, the effects of sociodemographic variables on the mean total times for the transport physical activity domain are inconclusive (Fig. 1.2).

### Nonparametric results for total weekly minutes of transport‐related physical activity

Participants working in the community and other areas of mental health (median = 150) scored higher on transport‐related physical activity than participants working in inpatient employment settings (median = 100) (Table 4). The Wilcoxon rank‐sum test *W*‐value was statistically significant (*W* = 7789, *P* = 0.003).

**Table 4:** Wilcoxon rank‐sum test results for total weekly minutes of transport‐related physical activity

Variable/Group	*n*	Median	IQR	Mean	Mean rank	*W* statistic	df	*P*‐value
Gender						5898	1	.380
Female	199	130	160	218	138			
Male	66	120	128	168	123			
Ethnicity						6514	1	.722
NZ European	164	120	130	192	128			
Others	103	130	180	225	144			
Area						7789	1	.003
Community & others	178	150	180	215	139			
Inpatient	89	100	102	180	123			

The Kruskal–Wallis test for total weekly minutes of transport‐related physical activity levels revealed that there was a significant difference of mean ranks (*H* (5) = 5.976, *P* = 0.050) between the age groups of 18–34 years, 35–54 years, and 55 years and above (Table 5). Post hoc analyses using the Wilcoxon rank‐sum test were then applied to test pairwise comparisons. The *P*‐value was adjusted using the Benjamini–Hochberg method to control for multiple comparisons.

**Table 5:** Kruskal–Wallis test results for total weekly minutes of transport‐related physical activity

Variable/Group	*n*	Median	IQR	Mean	Mean rank	H statistic	df	*P*‐value
Age						5.976	2	.050
18–34 years	45	120	105	205	126			
35–54 years	122	120	150	194	125			
55 years or above	100	160	190	216	149			
Registration						1.210	2	.546
0–5 years	57	130	120	208	129			
6–20 years	121	120	125	204	134			
21+ years	89	130	190	203	137			

The Wilcoxon rank‐sum test for total weekly minutes of transport in Table 6 showed none of the pairs were significant at the 5% level. The following two pairs were only marginally significant: 18–34 years compared with 55 years and above (*P* = 0.084) and 35–54 years compared with 55 years and above (*P* = 0.084).

**Table 6:** Pairwise comparisons using Wilcoxon rank‐sum test for total weekly minutes of transport‐related physical activity between age groups (*P*‐values)

18–34 years		35–54 years
35–54 years	0.844	
55 years or above	0.084	0.084

### Total mean minutes spent sitting

Figure 1.3 shows non‐significant differences in total weekly sitting time by gender.

### Nonparametric results for total weekly minutes of sitting behaviours

The Wilcoxon rank‐sum test showed no significant evidence of a difference in the mean rank scores on gender, ethnicity, and area categories for total weekly minutes spent sitting. (Table 7).

**Table 7:** Wilcoxon rank‐sum test results for total minutes spent sitting weekly

Variable/Group	*n*	Median	IQR	Mean	Mean rank	*W* statistic	df	*P*‐value
Gender						4451	1	.563
Female	199	600	420	725	135			
Male	66	590	375	726	128			
Ethnicity						5081	1	.252
NZ European	164	600	420	704	127			
Others	103	600	372	784	145			
Area						5324	1	.939
Community & others	178	600	420	741	133			
Inpatient	89	600	420	716	136			

The Kruskal–Wallis test for total weekly minutes spent sitting showed that there was a significant difference in the mean ranks between registration (*H*(2) = 8.694, *P* = 0.013) for 0–5 years, 6–20 years, and 21+ years (Table 8). A post hoc analysis using the Wilcoxon rank‐sum test was conducted to test pairwise comparisons. The *P*‐value was adjusted using the Benjamini–Hochberg method to control for multiple comparisons. The post hoc Wilcoxon rank‐sum test found that only one pair was significant for the registration group: 6–20 years compared with 21+ years (*P* = 0.02) (Table 9).

**Table 8:** Kruskal–Wallis test results for total minutes spent sitting weekly

Variable/Group	*n*	Median	IQR	Mean	Mean rank	H statistic	df	*P*‐value
Age						2.766	2	.251
18–34 years	45	660	450	723	143			
35–54 years	122	600	420	777	135			
55 years or above	100	540	400	681	129			
Registration						8.694	2	.013
0–5 years	57	660	375	704	136			
6–20 years	121	645	578	854	148			
21+ years	89	515	352	660	123			

**Table 9:** Pairwise comparisons using Wilcoxon rank‐sum test for total minutes spent sitting weekly between years of registration (*P*‐values)

	0–5 years	21+ years
6–20 years	0.38	0.02
21+ years	0.11	

### Total mean weekly minutes for all physical activities

The total minutes spent doing physical activities was estimated by calculating the sum of weekly time spent doing work‐related, transport‐related, household‐related, and recreation‐related physical activities. However, leisure and household physical activity levels were not specifically reported in this study. Survey responses to these questions were presented in relation to the demographic groups.

The bar plots of the mean total weekly minutes of all physical activities showed minor differences between mean values for each category: gender, age, ethnicity, area, and registration (Fig. 1.4).

### Nonparametric results for total weekly minutes of all physical activities

Participants working in the community or other areas of mental health (median = 465) scored higher than participants working in the inpatient areas of mental health (median = 400) (Table 10).

**Table 10:** Wilcoxon rank‐sum test results for total weekly minutes of all physical activities

Variable/Group	*n*	Median	IQR	Mean	Mean rank	*W* statistic	df	*P*‐value
Gender						5615	1	0.758
Female	199	440	510	600	134			
Male	66	510	362	592	137			
Ethnicity						7084	1	0.654
NZ European	164	440	418	585	130			
Others	103	490	518	612	140			
Area						7912	1	0.057
Community and others	178	465	432	597	139			
Inpatient	89	400	525	591	124			

The Wilcoxon rank‐sum test *W*‐value was closed to significance (*W* = 7912, *P* = 0.057).

The Kruskal–Wallis test for total weekly minutes of all physical activities showed a significant difference in the mean ranks of age (*P* = 0.003). Total minutes spent on all physical activities between the age groups (*H*(2) = 11.59, *P* = 0.003) included a median of 305 for age group 18–34 years, 450 for age group 35–54 years, and 550 for age group 55 years and above.

Post hoc analysis using the Wilcoxon rank‐sum test was conducted. The *P*‐value was adjusted using the Benjamini–Hochberg method to control for multiple comparisons (See Table 11).

**Table 11:** Kruskal–Wallis test results for total weekly minutes of all physical activities

Variable/Group	*n*	Median	IQR	Mean	Mean rank	H statistic	df	*P*‐Value
Age						11.59	2	0.003
18–34 years	45	305	355	434	99			
35–54 years	122	450	465	607	132			
55 years or above	100	550	520	658	152			
Registration						2.66	2	0.264
0–5 years	57	385		502	116			
6–20 years	121	450		608	136			
21+ years	89	480		631	141			

The pairwise comparisons using the Wilcoxon rank test for total weekly minutes of all physical activities in Table 12 shows only two pairs were significant: 18–34 years compared with 35–54 years (*P* = 0.044) and the second pair, 18–34 years compared with 55 years or above (*P* = 0.002).

**Table 12:** Pairwise comparisons using Wilcoxon rank‐sum test for total weekly minutes of all physical activities between age groups (*P*‐values)

18–34 years		35–54 years
35–54 years	0.044	
55 years or above	0.002	0.118

When weekly physical activity domains were calculated separately in the >150 min per week group, 38% of participants in work‐related and 44% of participants in transport‐related physical activity did not achieve the IPAQ minimum of 150 min per week for moderate‐to‐vigorous physical activity levels. While in the <150 min/week category, 62% of work‐related participants and 56% of transport‐related participants did not meet the IPAQ minimum requirements for moderate‐to‐vigorous physical activity levels. However, when each individual domain is added together for ‘total physical activities’, then only 10% of MHNs engage in <150 min per week group and 90% in the >150 min per week group (Table 13).

**Table 13:** Percentage of nurses not achieving standard of 150 min of physical activities weekly

Activity	<150 min		≥150 min	
	Count	Percentage	Count	Percentage
Total	25	10	225	90
Job‐related	88	62	55	38
Transport‐related	134	56	107	44
Leisure‐related	118	60	78	40
Household‐related	120	55	98	45

### Qualitative comments

The additional comments section in the IPAQ invited all participants to provide any comments they wanted to make. All (*n* = 71) comments were analysed, of which 51 responses were grouped into four themes. These included the following: barriers to physical activity, MHNs using physical activity as a coping strategy, type of physical activity, and physical activity self‐awareness. There were 20 comments excluded from the analysis that were non‐responses.

### Barriers to physical activity

Thirty participants identified barriers that affected their ability to engage in physical activities, including injury, exhaustion, tiredness, commute times to work, work sitting behaviours, weather, watching TV, physically unwell, emotional stress, holiday, walking <10 min, and lack of decent footwear (Table 14).

**Table 14:** Barriers to physical activity

Barriers to physical activity	Observations	Quotes
Work sitting behaviours	The highest reported barrier to physical activity was work sitting behaviours. Of the 71 participants, nine participants identified sitting behaviours at work being barriers to physical activity. Seven participants reported work sitting behaviour themes consistent with the two quotes One participant reported deliberately engaging in leisure exercise due to work sitting behaviours One participant also suggested a stand‐up desk due to work sitting behaviours	*Sat in front of computer for too long every day at work doing "busy work"* *Between sitting with clients & sitting at my desk doing paper work & filling in forms, my work in community mental health is very sedentary* *Very sedentary at work so try to make up for this outside of work by bike/train/walk commute, dog walking and gym daily* *I would like to get a stand‐up desk as my job entails a lot of sitting down*
Injuries	The second highest barrier to physical activity was ‘injuries’ Of the 71 participants, four participants reported injuries being a barrier to physical activity, consistent with these two quotes	*I have a current injury which precludes me from doing any vigorous exercise at present* *I currently have a knee injury so activity is limited*
Exhaustion and tiredness	The third highest barrier to physical activity was exhaustion or tiredness. Four participants identified exhaustion or tiredness consistent with the below quotes	*I’m too exhausted to do anything but sit* *It looks really bad when you write it out doesn't it? Lately though, I'm too worn out to attend the gym outside of work*

### Physical activity as a coping strategy

Two participants identified physical activity as their coping strategy, consistent with the quote below.

I have taken up running home from work most afternoons as a de‐stress. Also running at weekends

Great question, I go to the gym mon ‐ fri and love it, wakes me up and sets me up for the day

### Type of physical activity

Of 71 responses, four identified work physical activities including walking being the type of physical activity consistent with the quote below.

Work with an assertive community outreach team so are out and about either on foot or driving most days

I live rurally, have a life style block, my leisure time is horse riding. I travel 91 kms each way from home to work Mon‐Fri. I walk during work hrs between the community and in‐patient unit.

While my job tends to be sedentary my home life is anything but that with hours of daily chores [live on a lifestyle property] and am a very keen Nordic walker 8‐10 ks per exercise event as often as weather allows.

I work out a lot and am on my feet for at least 6 hours a day in the inpatient unit on average on the open ward it would be 7 and a half hours

### Physical activity self‐awareness

Of 71 responses, three identified that they needed to increase their physical activity levels consistent with the two quotes.

Wow, I'm not doing much will have to get out there and get moving

I actually need to move more and do more active things

This has highlighted for me that I need to move it move it!!

## Discussion

This study is the first to investigate the physical activity levels of registered MHNs in New Zealand and determine if they meet both the IPAQ and the World Health Organization recommended minimum levels of moderate‐to‐vigorous physical activity (IPAQ Group 2015; World Health Organization 2018).

The outcome of this study is critical in the context of the current physical inactivity pandemic. The MHN workforce is strategically placed to promote physical activity in their patients, especially if they consistently engage in health‐promoting physical activities themselves (Bakhshi *et al*. 2015; Happell *et al*. 2012).

In responses to work‐related physical activity, participants who reported greater than (>) 150 min/week group comprised 38% who met the IPAQ minimum requirements for moderate‐to‐vigorous physical activity levels of 150 min (IPAQ Group 2015), while, in the less than (<) 150 min/week category, 62% of job‐related participants did not meet the IPAQ minimum requirements for moderate‐to‐vigorous physical activity levels (IPAQ Group 2015). These findings confirmed previous studies, where approximately 50% of nurses did not meet moderate‐to‐vigorous exercise level recommendations (Chiou *et al*. 2014; McCarthy *et al*. 2018). Work‐related physical activity levels were significantly higher for participants working in inpatient units than in community and other areas. Benzo *et al*. (2021) also added nursing work in inpatient units is physically demanding, after their study investigated the work‐related physical activity levels and sedentary behaviour patterns of fifty six registered inpatient nurses. Participants wore an accelerometer and inclinometer (ActivPAL) which monitored the time participants spent sitting, standing, and walking. The study examined a total of 195 12‐hour work shifts. The study outcomes from the 195 consecutive 12‐h shifts were as follows.

- Sitting behaviours of nurses on average were 272 min (38%)
- Standing behaviours were 339 min (47%)
- Registered nurses walked an average of 101 min (14%) for a mean total of 8172 steps per 12‐hour shift.

Moreover, there was no significant difference in work‐related physical activities by gender or age. This result is at variance with McCarthy *et al*. (2018), who found increasing age to be a significant barrier.

For the transport‐related physical activity, 44% of participants in the >150 min/week group met the IPAQ minimum requirements for moderate‐to‐vigorous physical activity levels of 150 min (IPAQ Group 2015), while, in the < 150 min/week category, 56% did not meet the IPAQ minimum requirements. Transport‐related physical activity levels were significantly higher in the community and other areas than in inpatient employment settings. Hallal *et al*. (2012) identified a significant association between the participant’s age and their transport physical activity levels across 122 countries worldwide.

In the United Kingdom, Laverty *et al*. (2018) found the body mass index was 2.03kg/m2 lower for all female participants over 50 years of age who increased their use of public transport compared with those who did not. The World Health Organization (2020) identified 50% of people in Europe travelled less than 5Km distance by car and recommended such distance ought to be covered by walking or cycling.

The study did not find a significant difference between inpatient clinic and community and other areas for total weekly minutes spent sitting. More females than males self‐reported their weekly sitting behaviours; however, these differences were not statistically significant. Other researchers (Mayo *et al*. 2018; Mielke *et al*. 2018) have found physical inactivity to be marginally higher in females than males. Nurses in the 55 years or above group reported the highest total physical activity levels. Participants who had been registered for between 6 and 20 years reported the highest sitting behaviours. These findings are concerning as there is growing evidence of a greater risk of cardio metabolic disease and mortality with increased sedentary time (Ekelund *et al*. 2016; Prince *et al*. 2019). This is consistent with the findings from a cross‐sectional web‐based survey that received responses from 335 registered nurses and reported 34.1% participants were overweight, 23.4% were obese, and 80.1% were sitting for 3 or more hours per day (Ross *et al*. 2019).

Total weekly minutes of all physical activities were marginally significantly higher in participants working in the community or other areas than those in the inpatient group (*P* = 0.057), which has also been confirmed in research by Priano *et al*. (2018). The total weekly minutes of all physical activities showed a marginally significant difference for those 18–34 years compared with 35–54 years, and a significant difference for those 18–34 years compared with over 55 years. The total weekly minutes spent doing physical activities were higher among males than females, although this difference was not significant (*P* = 0.758). In contrast, several other studies (Mayo *et al*. 2018; Mielke *et al*. 2018) found that physical activity levels were significantly higher in females than males. The findings from this study were compatible with the hypothesis that over 50% of MHNs in New Zealand were not achieving 150 min of moderate‐to‐vigorous physical activity per week when each physical activity domain was examined separately.

However, when individual physical activity domains were combined, only 10% of participants spent less than 150 min on moderate‐to‐vigorous physical activity per week for the total physical activity domain.

The three highest barriers to physical activity were sitting behaviours at work, injuries, and exhaustion or tiredness. Previous studies also identified barriers preventing nurses from achieving moderate‐to‐vigorous occupational physical activity levels, including overtime, irregular shifts, stress, poor self‐care, limited access to exercise facilities, and decentralised nurses’ stations, resulting in nurses walking shorter distances (Reed *et al*. 2018; Ross *et al*. 2017; Torquati *et al*. 2017).

The World Health Organization has recently updated its guidelines to recommend that every individual should undertake at least 150–300 min of moderate physical activity or 75–150 min of vigorous physical activity per week or a calculated combination of moderate and vigorous physical activity (World Health Organization 2020). However, the 2018 World Health Organization guidelines have been used in this study because they were current at the time the study was conducted.

### Limitations

The most important limitation of this study was the low response rate, despite receiving 266 responses.

A factor that may have contributed to the low response rate was ‘survey saturation’ (McPeake *et al*. 2014). MHNs frequently receive emails inviting them to participate in online surveys.

A second limitation, related to self‐reporting, may have been bias due to possible ‘over reporting’ of physical activity levels. The analysis showed that data were skewed, with outliers, and some responses had exceeded ‘1500 min or more’, suggesting these physical activity levels were over‐reported. Previous studies using the IPAQ questionnaire also suspected ‘over reporting’ (Flannery *et al*. 2014).

## Relevance for Nursing Practice

Several studies have confirmed that nurses who actively participate in physical activity are more effective in promoting healthy practices in their patients (Bakhshi *et al*. 2015; Esposito & Fitzpatrick 2011). MHNs are well placed to proactively assess patients’ physical activity levels and if trained in counselling could support health promotion interventions that promote greater physical activity. Physical inactivity has been reported to be higher in mental health patients than in the general population (Happell *et al*. 2012; Rosenbaum *et al*. 2016),

Workplaces can enable nurses, themselves, to undertake more physical activity. Nurses should be empowered and motivated by managers to increase their physical activity, given the harmful impacts of physical inactivity, which includes NCDs and metabolic disease (Priano *et al*. 2018; das Merces *et al*. 2019). Access to exercise facilities and workplace health and wellness programmes should be available to nurses. Nurse leaders can implement job rotation shifts as a means to promoting health and well‐being.

## Conclusion

This study addressed a gap in the literature and provided important insight into the physical activity levels in MHNs in New Zealand. However, a low response rate and statistical outliers suggest the need for caution in generalizing study findings to the whole MHN population. Key barriers to physical activity in this population were consistent with the literature. More in‐depth qualitative studies should be undertaken to identify strategies to mitigate barriers to physical activity.
